# Supplementary material for: Game analysis on PPP model operation of abandoned mines ecological restoration under the innovation of central government’s reward and punishment system in China
Source: PLoS One. 2024 May 29;19(5):e0304368. doi: 10.1371/journal.pone.0304368 (PMC11135726; doi:10.1371/journal.pone.0304368)
Supplement: S1 Appendix — (DOCX) [file pone.0304368.s001.docx]

## Appendix A

Proof of Proposition 2: Based on the equation of the two-dimensional dynamical system *I* given in Section 4.1, the determinant det*J* and trace tr*J* values of the four equilibrium points (0,0), (0,1), (1,0) and (1,1) in the Jacobian matrix can be first obtained in Section 4.2, then, according to Friedman's theory, the local stability of each equilibrium point can be judged. Here is the proof of the equilibrium point in Scenario 1 of Proposition 2 as follows:

From conditions $L_{g}<C_{h}-C_{d}<F_{e}+L_{g}\text{+}J_{e}$and$V_{g}+F_{e}+J_{g}<C_{g}-F_{g}$, it can be inferred that:

When the equilibrium point is (0, 0), there are$A_{11}=C_{d}-C_{h}+L_{g}<0$,$A_{12}=0$,$A_{21}=0$, $A_{22}=V_{g}-C_{g}\text{+}F_{g}+F_{e}<0$, then $TrJ=A_{11}+A_{22}<0$and$DetJ=A_{11}A_{22}-A_{12}A_{21}>0$, therefore the equilibrium point (0, 0) is the Evolutionary Stable Strategy Point (ESS).

When the equilibrium point is (1, 0), there are$A_{11}=-\left(C_{d}-C_{h}+L_{g}\right)>0$,$A_{12}=0$,$A_{21}=0$, $A_{22}=V_{g}+J_{g}-C_{g}<0$, then $TrJ=A_{11}+A_{22}<0 or>0$and$DetJ=A_{11}A_{22}-A_{12}A_{21}<0$, therefore the equilibrium point (1, 0) is the saddle point of the evolutionary strategy.

When the equilibrium point is (0, 1), there are$A_{11}=F_{e}+L_{g}+J_{e}\text{+}C_{d}-C_{h}>0$,$A_{12}=0$,$A_{21}=0$, $A_{22}=\text{-}\left(V_{g}-C_{g}\text{+}F_{g}+F_{e}\right)>0$, then $TrJ=A_{11}+A_{22}>0$and$DetJ=A_{11}A_{22}-A_{12}A_{21}>0$, therefore the equilibrium point (0, 1) is the unstable point of the evolutionary strategy.

When the equilibrium point is (1, 1), there are$A_{11}=\text{-}\left(F_{e}\text{+}L_{g}\text{+}J_{e}\text{-}C_{h}\text{+}C_{d}\right)<0$,$A_{12}=0$,$A_{21}=0$, $A_{22}=\text{-}\left(V_{g}\text{+}J_{g}\text{-}C_{g}\right)>0$, then $TrJ=A_{11}+A_{22}<0 or>0$and$DetJ=A_{11}A_{22}-A_{12}A_{21}<0$, therefore the equilibrium point (1, 1) is the saddle point of the evolutionary strategy.

The stability proof of the equilibrium point under this condition in Scenario 1 has been completed above, and the stability proof method for equilibrium points in other scenarios is the same way.
