# Supplementary material for: Game analysis on PPP model operation of abandoned mines ecological restoration under the innovation of central government’s reward and punishment system in China
Source: PLoS One. 2024 May 29;19(5):e0304368. doi: 10.1371/journal.pone.0304368 (PMC11135726; doi:10.1371/journal.pone.0304368)
Supplement: Attachment — Submitted filename: Response to Reviewers.docx [file pone.0304368.s003.docx]

## List of Actions

**LOA1:** Double line spacing and red text highlighting were applied to the modified content in the revised manuscript. The following modifications are explained in the order they appear in the revised manuscript.

**LOA2:** In consideration of feedback from multiple reviewers, the authors have rewritten the original manuscript's introduction. Integrating the comments from the reviewers, this section has been delineated into Introduction and Literature Review, with the latter now presented as a standalone section. Please refer to Sections 1 and 2 of the revised manuscript for detailed information on this segment.

**LOA3:** In the last section of the literature review in Section 2 of the revised manuscript, an explanation of the innovative points and characteristics of our research has been added. Please refer to the revised content in this section.

**LOA4:** In Section 3 of the revised manuscript, the hypothesis section has been rewritten, and the hypothesis and logical explanation of the hypothesis have been described separately to achieve the goal of making the premise hypothesis concise.

**LOA5:** Revised and corrected Proposition 2 in Section 4 of the revised manuscript, and supplemented the content of Table 3 attached to Proposition 2.

**LOA6:** Proposition 2 in Section 4 of the revised manuscript has been revised and corrected, and the parameters for Scenario 2 and Scenario 5 in Section 5 have been revised and adjusted. Figures 2 and 3 have been recalculated and revised.

**LOA7:** Proposition 2 in Section 4 of the revised manuscript has been proven, and the proof for this section has been presented in the form of an appendix at the end of the revised manuscript.

**LOA8：**At the end of Section 6 of the revised manuscript, an explanation of possible research directions for future research was added.

**LOA9：**All typos have been removed.

## Responses to Editor

We have double spaced the text throughout the revised manuscript, see LOA1.

## Responses to Reviewers

We are grateful to reviewer for his/her effort reviewing our paper and his/her positive feedback. The summaries of our work as written by these reviewers are precise. Here below we address the questions and suggestions raised by the reviewer 2 and 3. We have carefully addressed all the reviewer's concerns. Please see below our replies. We hope he/she is satisfied with our answers and the new (figures or data) we provided. Changes highlighted in red and double spaced the text have been made accordingly in the revised manuscript.

### To Reviewer 1:

Thank you to this reviewer for their summary and recognition of our work, as well as for their efforts and positive feedback during the review of our paper.

### To Reviewer 2:

***Opinion 1:*** *In this paper, an evolutionary game model of "local government-social capital" was developed to analyze the interaction and behavior mechanism of core stakeholders in the operation process of abandoned mines ecological restoration PPP mode. It has some significance. However, J have the following concerns*

*1) --Literature Review should be presented as a separated section. Essentially, many papers have examined the evolutionary game .*

*For example：*

*--Zhang S., Wang, C.X., Yu C., The evolutionary game analysis and simulation with system dynamics of manufacturer's emissions abatement behavior under cap-and-trade regulation, Applied Mathematics and Computation, 2019, 355, 343-355.*

*--Shi Y., Wei Z., Shahbaz M., Zeng Y., Exploring the dynamics of low-carbon technology diffusion among enterprises: An evolutionary game model on a two-level heterogeneous social network, 2021,101: 105399.*

*--Jiang N., Feng Y., Wang X., Fractional-order evolutionary game of green and low-carbon innovation in manufacturing enterprises, Alexandria Engineering Journal, 2022,61(2), 12673-12687.*

*It is suggested that author(s) introduce the papers related to the above evolutionary game analysis into the literature review, and identify the differences between the previous papers and current paper. Thus, the authors have to differentiate further and rethink about their work's innovations.*

**Argue and Revise:** Thank you for pointing this out. Regarding the review section in the introduction of the paper, the authors have undertaken a substantial revision. In accordance with the recommendations of the reviewers, the review section has been crafted into an independent chapter. Furthermore, based on the detailed suggestions of another reviewer, adjustments have been made to the narrative logic, as outlined in LOA2. The revised structure involves (1) a review of traditional approaches leading to the proposal of the PPP model, (2) a concise overview of the PPP model and related studies, and finally, (3) an exposition of research gaps and the introduction of the innovative aspects of our paper. In the section reviewing the PPP model, we have consulted and cited literature provided by the reviewers. We have elaborated on the latest developments in the integration of evolutionary game models across various domains, drawing inspiration for our paper. Additionally, we have argued evolutionary game models to address the issue of interest distribution among multiple stakeholders in PPP relationships, delving into the specific realm of environmental governance in mining projects. We have discussed the progress and challenges in this field, incorporating insights from the literature review and concluding with the presentation of our research's innovative contributions and distinctive features. Please refer to LOA2 in the revised manuscript for a detailed account of these modifications.

***Opinion 2:*** *The innovation points for this paper should be clearly given in Introduction.*

**Revise:** Thank you for pointing this out. The authors have undertaken a revision of this section, and in the final part of the revised manuscript's second chapter - the literature review, they have provided an explanation of the innovative aspects and distinctive features of their research. Please refer to the modified content in that section for further details, see LOA3.

***Opinion 3:*** *In Section 4 Numerical simulation analysis , where the initial values of the parameter comes? Explanations of the basis for setting the values of these parameters should be presented.*

**Argue and Reply:** Thank you for pointing this out. The study investigates the strategic interactions and adjustments between government regulatory agencies and private sectors, employing Matlab simulation to analyze the evolutionary impact of equilibrium strategies and model parameter adjustments on the strategic choices of both parties in different scenarios. The aim of the paper is to abstract key variables for analyzing the various equilibrium relationships formed from the game dynamics between the two parties in PPP projects. This provides a more intuitive understanding of the dynamic evolution trends in the quality supervision process of ecological restoration in mining under PPP operations. Based on the conclusions from the analysis, the paper suggests measures to enhance the service quality of the PPP operational model for mining ecological restoration from the perspective of government regulation. The focus of this article is primarily on simulation analysis and has limited connection with statistical analysis from actual cases. In the revised manuscript's Section 5(Section 4 in origin manuscript), which meets the parameter requirements of Assumption 2 in Section 4, different scenarios for the operational model of mining ecological restoration PPP can be assigned based on project-specific conditions, and the values chosen do not represent the actual costs or benefits for government regulatory agencies and private capital in mining ecological restoration PPP projects. If there are actual cases of PPP projects for mining environmental restoration with operational data, the approach presented in this paper can be utilized to validate and retrospectively analyze real projects from the perspective of game theory, shedding light on the reasons for project success or failure.

***Opinion 4:*** *In Section 5. Conclusions and suggestions，the future research directions should be presented.*

**Revise:** Thank you for pointing this out. The authors have supplemented this section based on the suggestions of the reviewers. Please refer to the final section of Chapter 6 of the revised manuscript, see LOA8.

### To Reviewer 3:

***Opinion 1:*** *The narrative order about the literature review needs to be adjusted. The authors focus on the ecological restoration of abandoned mine from the perspective of an economic approach, so after providing the background information, the authors should review the traditional perspectives and approaches in the previous researches on the ecological restoration of abandoned mine and then put forward the PPP model given that this topic lacks an economic perspective. The brief review of the PPP model and the researches with it should be presented after putting forward the PPP model. When finishing the above work, the authors need to elaborate “However, the ecological environment restoration of mines generally lacks a good income mechanism, and social capital is still in the wait-and-see and exploration stage at present”. Based on this work, “it is of great research value to explore the application of PPP model innovation in low-profit mine ecological restoration projects” can be better understood. So how to adjust the Introduction is clear. The first and last paragraph need not to be adjusted. The middle content can be conducted as follows: (1) Review the traditional approaches and put forward the PPP model; (2) Brief review the PPP model and the researches with it; (3) Elaborate the research gap.*

**Revise and Reply:** Thank you for pointing this out. Regarding the review section of the paper, the authors have rewritten it. According to the reviewer's suggestions, adjustments have been made to the narrative logic in this revised section of the paper. And based on the suggestions of another reviewer, this section separates the overall cutting-edge content into two separate sections, namely the introduction and literature review. The details of the modifications can be found in the description of this section of the revised manuscript, see LOA1.

***Opinion 2:*** *The shortcomings (and contributions if they are important) in the relevant literature should be clear instead of “someone did something, some did something…”.*

*The authors can criticize the previous researches from method or evidence to highlight the research value.*

**Revise and Reply:** Thank you for pointing this out. The content of this section has been rewritten, and based on the suggestions of the reviewers, the narrative logic has been modified and comments on previous literature have been added. For more details, please refer to the modifications made to this section in the revised manuscript. The citations of previous literature are reflected in the narrative logic of each section. For example, in the review of the PPP model section, we read and cited the literature materials provided by the reviewers, and elaborated on and explained the latest research situation of the combination of evolutionary game models in multi domain practice, in order to provide us with inspiration points and innovative possibilities for transplanting to a new field in our paper. In addition, The application of evolutionary game models in the study of the distribution of interests among multiple stakeholders involved in public-private partnerships such as PPP is explored. This paper delves into the special field of mining environmental governance projects, as well as the progress and existing problems in this field of research. At the end of the literature review, innovative points and characteristics of my own research are proposed, as shown in LOA1.

***Opinion 3:*** *The hypotheses should be concise. The authors can explain the logic of a hypothesis before presenting the hypothesis. Do not put the hypothesis and its explanation together.*

**Revise and Reply:** Thank you for pointing this out. We have made modifications to this section based on the comments of the reviewers. Please refer to Section 3 of the revised manuscript for details, see LOA4.

***Opinion 4:*** *Why choose the evolutionary game model to explain the operation of PPP projects? or how the operation of PPP projects manifests the characteristics of evolutionary games? The authors need to expound the connection between the evolutionary game model and the operation of PPP projects, or summarize several stylized facts of PPP projects, which are in accord with the characteristics of evolutionary games. This work can be put in the Introduction or Model hypothesis and construction.*

**Argue and Revise:** Thank you for pointing this out. The authors have seen that in the operation of the PPP project for ecological restoration of abandoned mines, whether from the perspective of risk sharing and profit distribution, or from the regulatory perspective, there are game relationships among all parties to ensure the smooth progress of the PPP project. Starting from this idea, the authors gradually rationalize the operating mechanism of PPP in this field, analyze the contractual relationship and project participants in the PPP project for ecological restoration of abandoned mines, and construct a game framework for both government public sectors and social capital investors. The authors have rewritten this section based on the comments of the reviewers, detailing the content of Section 1 and 2 of the revised manuscript. The authors have reflected these narrative logics in the revised manuscript, for example, in the review of the PPP model section, explaining the latest research on the combination of evolutionary game models in multi-domain practice, as well as the various applications of evolutionary game models in PPP projects. Finally, they delve into the special field of mining environmental governance projects, as well as the progress and existing problems in this field of research. Finally, they propose their own innovative points and characteristics in the literature review, see LOA2.

***Opinion 5:*** *Given that the PPP model has been conducted in reality, how do the authors interpret if the actual operation does not reach to “equilibrium”? Consider giving some possible reasons.*

**Argue and Reply:** Thank you for pointing this out. The dynamic evolutionary game in the paper is a benefit matrix obtained by assuming conditions, and finally a replicated dynamic equation is obtained. Through the analysis of equilibrium points and evolutionary strategy stability, 5 equilibrium points of the system are obtained. These 5 equilibrium points are analyzed and proven in sections 4.1 and 4.2. Through a detailed analysis of these equilibrium points, it can be concluded that different project parameters are more favorable to the government in some cases and more favorable to the social capital in others. From the basic game logic, we hope that this game equilibrium can achieve the optimal effect, that is, both parties can profit the most. Therefore, we have redefined some more optimized combination conditions to meet this goal. In addition, Section 5 of the revised manuscript also mentions "Scenario 2", in which the system does not have an evolutionary stable strategy, that is, it cannot reach an evolutionary equilibrium strategy. According to Friedman's theory, the stability of Scenario 2 is analyzed in Table 3, and the reason for this instability is explained through numerical simulation in Section 5.1. For detailed explanation, please refer to "Scenario 2" in Section 5.1. Under the condition of Scenario 2, the behavior evolution of government regulatory agencies and social capital is in a cyclical swing state, and will not form or reach a stable evolution strategy, that is, cannot reach an equilibrium state between the two. This also explains some states from a practical perspective: during the high incidence period of ecological restoration service quality issues in mines in China, most government regulatory departments adopt an active and active regulatory model in pursuit of maximizing social benefits. However, during the low incidence period of accidents, in order to ensure the interests of their own departments, the extremely intermittent regulatory model is abolished, The central government's commitment to reward local governments and social capital cannot serve as a stable strategic choice for both sides, similar to a game of cat and mouse turning trap.

**Opinion 6:** *The proofs need to be presented in details. The authors can put the detailed process in the appendix. Otherwise, we can hardly understand the game result without patience and enthusiasm.*

**Revise and Reply:** Thank you for pointing this out. The authors have supplemented this section based on the suggestions of the reviewers. Please refer to the appendix of the revised manuscript. And supplemented Table 3 of the original manuscript. We have also revised some of the issues in the original manuscript and made some adjustments in the later calculation section. Please refer to Section 5 of the revised manuscript for details, see LOA5, LOA6 and LOA7.
