# Supplementary material for: Game analysis on PPP model operation of abandoned mines ecological restoration under the innovation of central government’s reward and punishment system in China
Source: PLoS One. 2024 May 29;19(5):e0304368. doi: 10.1371/journal.pone.0304368 (PMC11135726; doi:10.1371/journal.pone.0304368)
Supplement: Attachment — Submitted filename: Response to Reviewers.docx [file pone.0304368.s004.docx]

## List of Actions

**Statement:** Red text highlighting were applied to the modified content in the revised manuscript. The following modifications are explained in the order they appear in the revised manuscript.

**LOA1:** In order to make the model in this article more applicable, the authors have revised and rewritten the result analysis of Scenario 2 in Section 5.1 based on the comments of the reviewer, and cited some literatures on stylized failure cases analysis in PPP projects to more effectively and clearly manifest the actions of the governments.

## Responses to Editor

We have highlighted the revised text in red throughout the entire revised manuscript, please refer to the Statement.

## Responses to Reviewers

We are grateful to reviewer for his/her effort reviewing our paper and his/her positive feedback. The summaries of our work as written by these reviewers are precise. Here below we address the questions and suggestions raised by the reviewer 3. We have carefully addressed all the reviewer's concerns. Please see below our replies. We hope he/she is satisfied with our answers and the new (figures or data) we provided. Changes highlighted in red have been made accordingly in the revised manuscript.

### To Reviewer 3:

***Reviewer #3:*** *Reconsider the "Option 4" and "Argue and Revise". What are the possible reasons if the actual operation does not reach to “equilibrium”? Given that China has a large number of administrative units, you can analyze why some administrative units adopt a kind of regulation and why the others administrative units do not adopt this kind of regulation during the different incidence period. The authors' response, "during the high incidence period of ecological restoration service quality issues in mines in China, most government regulatory departments adopt an active and active regulatory model in pursuit of maximizing social benefits. However, during the low incidence period of accidents, in order to ensure the interests of their own departments, the extremely intermittent regulatory model is abolished", just gives the theoretical reasons or the theoretical assumptions, ignores the reality explanations. To make the model in this paper more applied, the authors can give some stylized facts or cases that clearly manifest the actions of the governments.*

*Whether to modify this issue depends on the author's wishes.*

**Argue and Reply:** Thank you for pointing this out. The authors apologize that their response to this question may not have been clear and thorough enough before. The previous response to this question provided a theoretical and detailed explanation. Please refer to the previous detailed response to this question, and it also provided an explanation for a typical programmatic failure situation of the government in the supervision of many PPP projects in reality, that is, most governments adopt a movement style supervision mode in pursuit of maximizing social benefits, which is consistent with the description of the model's Scenario 2 results, and this is also a typical failure situation in many PPP project supervision cases.

The authors provide the following explanations and revisions to the questions raised by the reviewer:

Firstly, reply to the question: *Given that China has a large number of administrative units, you can analyze why some administrative units adopt a kind of regulation and why the others administrative units do not adopt this kind of regulation during the different incidence period.*

The two objects studied in the paper (government departments and social capitals) are both groups that change over time. The factors that affect the changes of these two groups have both regularity presented through the selection process during evolution (the selection process has a certain inertia), and at the same time, this process also harbors the driving force of mutation. Mutation is a learning and imitation process that constantly trial and error. When all participating entities in the game between government departments and social capitals adopt the same behavior strategy respectively, such as when the behavior strategy of certain entities in the government department group undergoes a sudden change (such as when facing "good service" provided by certain social capital, the government department chooses to negative supervision), if the benefits brought by the behavior strategy of that entity are higher than those of other entities in the group, then other participating government department entities will also follow the behavior strategy of the mutated entity and adopt similar behavior strategies. As a result, the proportion of individuals adopting the new strategy in the entire game group will increase. As the game progresses and time passes, the behavior strategy with higher returns will gradually replace the behavior strategy with lower returns, the individual experience of government departments or social capital adopting this strategy is increasing, but there will be some inertia in the game process that makes the evolutionary game process impossible to achieve instantaneously. That is, it will not happen immediately. All government departments or social capital will adopt the same strategy at the same time, and there is a process of change. Therefore, in different periods of occurrence, some administrative units will adopt certain regulations, while other administrative units will not adopt such regulations. In addition, Section 5.1 of the paper also mentions "Scenario 2", in which the system does not have an evolutionarily stable strategy, that is, it is impossible to achieve an equilibrium strategy of evolution. Under the conditions of "Scenario 2", the behavior evolution of government departments and social capitals are in a cyclical swing state, and it will not form or reach a stable evolution strategy, that is, it will not reach an equilibrium state between the two groups, and a cyclical game strategy cycle will be formed between the two groups.

Secondly, reply to the question: *just gives the theoretical reasons or the theoretical assumptions, ignores the reality explanations. To make the model in this paper more applied, the authors can give some stylized facts or cases that clearly manifest the actions of the governments.*

In actual PPP project implementation, project implementation failures often occur, and a typical stylized case is the game of "cat and mouse" that often occurs in government departments and social capitals in supervision. In China, during the high-incidence period of ecological restoration service quality problems in mines, most government departments adopt an active regulatory model in pursuit of maximizing social benefits. In the low-incidence period of accidents, in order to ensure the interests of their own departments, negative regulatory models are adopted. The authors have made modifications to this part and correspondingly cited some stylized facts or case analysis results as evidence. The model established in this article simulates Scenario 2, which provides a good explanation for this imbalance in reality. Detailed modifications can be found in LOA1.
